# Dose Dependency of Iatrogenic Glucocorticoid Excess and Adrenal Insufficiency and Mortality: A Cohort Study in England

**DOI:** 10.1210/jc.2019-00153

**Published:** 2019-04-22

**Authors:** Teumzghi F Mebrahtu, Ann W Morgan, Adam Keeley, Paul D Baxter, Paul M Stewart, Mar Pujades-Rodriguez

**Affiliations:** 1 Leeds Institute of Biomedical and Clinical Sciences, School of Medicine, University of Leeds, Leeds, United Kingdom; 2 Leeds Institute of Cardiovascular and Metabolic Medicine, School of Medicine, University of Leeds, Leeds, United Kingdom; 3 NIHR Biomedical Research Centre, Leeds Teaching Hospitals NHS Trust, Chapel Allerton Hospital, Leeds, United Kingdom; 4 Leeds Institute of Data Analytics, University of Leeds, Leeds, United Kingdom; 5 Dean’s Office, Faculty of Medicine & Health, University of Leeds, Leeds, United Kingdom; 6 Leeds Institute of Health Sciences, School of Medicine, University of Leeds, Leeds, United Kingdom

## Abstract

**Context:**

Adrenal insufficiency and Cushing syndrome are known adverse events of glucocorticoids. However, no population estimates of dose-related risks are available.

**Objective:**

To investigate dose-related risks of adrenal dysfunction and death in adults with six chronic inflammatory diseases treated with oral glucocorticoids.

**Design and setting:**

Retrospective, record-linkage, open-cohort study spanning primary and hospital care in England.

**Patients:**

A total of 70,638 oral glucocorticoid users and 41,166 nonusers aged ≥18 years registered in 389 practices in 1998 to 2017.

**Main outcome measures:**

Incidence rates and hazard ratios (HRs) of diagnosed adrenal dysfunction and death.

**Results:**

During a median follow-up of 5.5 years, 183 patients had glucocorticoid-induced adrenal insufficiency and 248 had glucocorticoid-induced Cushing syndrome. A total of 22,317 (31.6%) and 7544 (18.3%) deaths occurred among glucocorticoid users and nonusers, respectively. The incidence of all outcomes increased with higher current daily and cumulative doses. For adrenal insufficiency, the increases in HRs were 1.07 (95% CI: 1.04 to 1.09) for every increase of 5 mg per day and 2.25 (95% CI: 2.15 to 2.35) per 1000 mg of cumulative prednisolone-equivalent dose over the past year. The respective increases in HRs for Cushing syndrome were 1.09 (95% CI: 1.08 to 1.11) and 2.31 (95% CI: 2.23 to 2.40) and for mortality 1.26 (95% CI: 2.24 to 1.28) and 2.05 (95% CI: 2.04 to 2.06).

**Conclusion:**

We report a high glucocorticoid dose-dependent increased risk of adrenal adverse events and death. The low observed absolute risk of adrenal insufficiency highlights a potential lack of awareness and a need for increased physician and patient education about the risks of adrenal dysfunction induced by glucocorticoids.

## Teaser

In this cohort study, we found high glucocorticoid dose-dependent increased risks of adrenal dysfunction and death but low absolute risks, indicating potential underdiagnosis in clinical practice.

Glucocorticoids are widely used for the treatment of chronic inflammatory diseases and are administered through a variety of routes including oral, inhaled, and topical as well as systemic therapies (1–3). In 2008, it was estimated that 0.8% of the UK adult population had used glucocorticoids for 3 months or more, rising to 3% in women older than 80 years (4). Glucocorticoids are effective in controlling underlying disease inflammation and reducing symptoms in the majority of patients with chronic inflammatory diseases. However, their continuous use can cause cushingoid features in the context of exogenous Cushing syndrome with circulating glucocorticoid excess (5, 6). Paradoxically, adrenal insufficiency can also occur because the glucocorticoid excess suppresses endogenous cortisol secretion by negative feedback upon the hypothalamo-pituitary-adrenal axis (7–9).

Previous studies have reported absolute risk estimates for adrenal insufficiency (1, 8, 10) and Cushing syndrome (11) in adults treated with glucocorticoids. For example, a recent meta-analysis including 3753 individuals receiving glucocorticoid therapy from 36 clinical trials and 38 observational studies reported an absolute risk of adrenal insufficiency of 31.7% (48.7% in patients treated with oral glucocorticoids) (1). Most patients had atopic diseases (*e.g.,* asthma, rhinitis, dermatitis), cancer, or organ transplantation, with studies including short, medium, and long-term glucocorticoid users.

The long-term use of glucocorticoids has also been associated with a high risk of mortality (12), with many of the deaths caused by the underlying disease being treated. However, no estimates of dose-related risk of adrenal adverse events or mortality are available to guide and evaluate clinical practice. The aim of this population-based study was to investigate dose-related risks of adrenal insufficiency, Cushing syndrome, and death in people with underlying chronic inflammatory diseases commonly treated with oral glucocorticoids.

## Materials and Methods

### Study population

The study was conducted among patients registered in general practices of the Clinical Practice Research Datalink (CPRD) who had consented to data linkage between 1 January 1998 and 15 March 2017. Eligibility criteria for study inclusion were a minimum of 1 year of registration in the practice, age 18 years or older, and a diagnosis of inflammatory bowel disease, systemic lupus erythematosus, polymyalgia rheumatica, giant cell arteritis, rheumatoid arthritis, and/or vasculitis before or during the study period.

Patient data from three linked data sources spanning primary and hospital care were used. Primary health care records from the CPRD (13) were used to identify diagnoses of diseases (*e.g.,* adrenal insufficiency), prescribed medication, and results of laboratory tests. Hospital records from the Hospital Episode Statistics (www.hscic.gov.uk/hes) were used to identify diagnoses recorded during hospitalization. Data from the Office for National Statistics (https://www.ons.gov.uk/atoz?query=mortality&size=10) were used to obtain information on the index of multiple deprivation (14) and to identify dates and causes of death. Codes used to identify patients with each of the chronic inflammatory diseases are shown in an online repository (15).

### Study design

This is a retrospective, population-based, record-linkage cohort study. For each patient, the start of follow-up was the earliest date on which all the eligibility criteria were met. Follow-up ended on the date of first occurrence of the outcome analyzed (adrenal insufficiency, Cushing syndrome, or death), de-registration from the practice, or last date of data collection in the practice, whichever occurred first. Patients were divided into two groups, glucocorticoid users and nonusers, according to whether at least one prescription of glucocorticoids was issued to the patient in the period between 1 year prior to study start and the end of follow-up.

### Outcome variables

The primary outcomes were glucocorticoid-induced adrenal insufficiency and Cushing syndrome, which were assessed in the glucocorticoid user population. All-cause mortality was a secondary outcome, and it was evaluated among glucocorticoid users and nonusers.

Algorithms for adrenal insufficiency and Cushing syndrome case identification were developed to minimize misclassification of outcome status (15). Patients with one or more diagnostic codes recorded in any of the three data sources and who received oral glucocorticoids within 1 year prior to the diagnosis but no other drugs that might have influenced function of the hypothalamo-pituitary-adrenal axis (*e.g.,* mifepristone and megestrol acetate) in the previous 6 months were considered to have the outcome of interest. Lists of Read and International Classification of Diseases (versions 9 and 10) codes for adrenal insufficiency and Cushing syndrome diagnoses and medications used in the algorithms can be found in the online repository (15).

**Figure 1.**
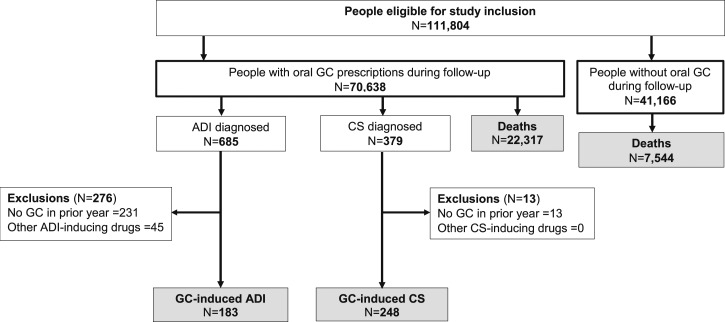
Study flow diagram and outcome identification. Eligibility inclusion criteria were a minimum of 1 year of registration in the CPRD practice, consent to data linkage, age ≥18 years, and a diagnosis of inflammatory bowel disease, systemic lupus erythematosus, polymyalgia rheumatica, giant cell arteritis, rheumatoid arthritis, and/or vasculitis before or during the study period (1 January 1998 to 15 March 2017). During periods of nonglucocorticoid exposure, 102 patients had adrenal insufficiency and 94 patients had Cushing syndrome diagnosed at a median of 52 [interquartile range (IQR): 31 to 79] d and 42 (IQR: 30 to 63) d, respectively, following glucocorticoid discontinuation. ADI, adrenal insufficiency; CS, Cushing syndrome; GC, glucocorticoid.

Figure 1 presents the flowchart of the study and the number of patients diagnosed with each study outcome.

### Oral glucocorticoid exposure

Exposure status was defined using drug prescriptions for any type of oral glucocorticoids issued in primary care. Current and cumulative doses were derived from the recorded product strength (*e.g.,* 5 mg), directions given (*e.g.,* twice daily), and prescribed quantity (*e.g.,* 100 tablets). Because the relative anti-inflammatory effects of different types of glucocorticoids vary, dosages were converted into prednisolone-equivalent doses, with 10 mg of prednisolone considered to be equivalent to 0.96 mg of beclomethasone, 1.09 mg of budesonide, 1.5 mg of betamethasone and dexamethasone, 8 mg of methylprednisolone and triamcinolone, 10 mg of prednisone, 12 mg of deflazacort, 40 mg of hydrocortisone, and 50 mg of cortisone (16).

To minimize length- and time-dependent bias (17), patient exposure status was determined by taking into account periods without prescribed medication. For example, the period between the start of follow-up and the first recorded oral glucocorticoid prescription (*i.e.,* time at risk) and the periods not covered by prescribed medication were classified as unexposed. The end date of each drug prescription was not available and was calculated by adding the duration of days covered by the prescription to the date when the prescription was issued. For each prescription recorded, its duration was calculated as the number of tablets prescribed divided by the daily dose. When data on the daily dose or the number of tablets prescribed were missing, they were imputed using truncated regression (18). The number of data sets to be imputed was assumed to be at least 100 times the fraction of incomplete cases (19). Hence, a total of 20 data sets were imputed. Variables included for imputation were nonoral glucocorticoid prescription during follow-up, sex, baseline comorbidities (hypertension, cardiovascular disease, and diabetes), and duration of the first recorded underlying chronic inflammatory disease. The imputed values of daily dose and number of tablets prescribed were then averaged to replace the missing values of each variable before estimating the end date of each prescription and calculating the survival time. This approach was adopted given that the missing values in the two variables would affect the survival time and that the incidence of the adrenal outcomes was very low (0.3% to 0.4%), which would have biased the Nelson-Aalen estimator and event indicator variables (20).

**Figure 2.**
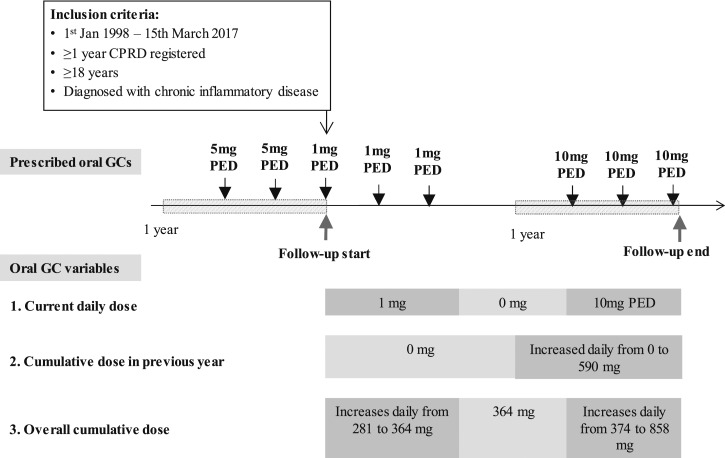
Definition of time-variant oral glucocorticoid exposure variables. Inclusion criteria included a diagnosis of inflammatory bowel disease, systemic lupus erythematosus, polymyalgia rheumatica, giant cell arteritis, rheumatoid arthritis, and/or vasculitis before or during the study period (1 January 1998 to 15 March 2017). GC, glucocorticoid; PED, prednisolone equivalent dose.

Three types of time-variant oral glucocorticoid exposure variables were initially defined (Fig. 2): current daily dose [*i.e.,* the value corresponding to the dose prescribed for the duration covered by the prescription and changed on the date(s) on which the prescribed dose increased or decreased]; cumulative dose in the previous year (*i.e.,* the value was “zero” until 1 year before the end of follow-up; from that date, it corresponded to the cumulative prescribed dose during the last year of follow-up); and cumulative dose since 1 year prior to study entry (*i.e.,* the value corresponding to the cumulative prescribed dose until a given time point and changed with the daily dose prescribed to the patient during the time of follow-up). Ordinal and continuous variables were considered in the analyses, with cutoff points at 0, 5, and 7.5 mg for current daily dose and at 0, 960, and 3055 mg for cumulative dose variables. These cutoffs were based on previous studies (12) to facilitate comparisons.

### Confounding variables

Confounding selection was guided by the epidemiological, biological, and clinical understanding of the authors. To minimize bias due to confounding and overadjustment, directed acyclic graphs were used (21, 22) to select confounding variables, and models were tested using DAGitty software (23). This method identified nonoral glucocorticoids (inhaled, intra-articular, parenteral, nasal, topical, and rectal routes) as the “minimal sufficient” set of adjustment variables (15). For mortality, baseline asthma, cancer, heart failure, chronic kidney disease (stages 4 or 5), time-variant chronic inflammatory diseases, and prescribed nonoral glucocorticoids during follow-up were considered confounders (15).

### Statistical analysis and software

Dose-specific incidence rates were calculated as the number of events divided by the total number of person-years at risk. The incidence ratios of death for patients with adrenal insufficiency and Cushing syndrome, compared with those for oral glucocorticoid user and nonuser populations, were also estimated. Furthermore, Kaplan-Meier dose-related risks of outcomes were reported at 5 and 10 years.

Cox proportional hazard models (24) were used to estimate the risk of glucocorticoid dose-related adrenal dysfunction and mortality. Adjusted and unadjusted models were constructed for each outcome. Tests of proportional-hazards assumptions were implemented by interacting the analysis time with exposure and confounding variables and verifying that the interaction coefficients were not different from zero (25). Model convergence was assumed if the best log-likelihood (global maxima) was achieved. The Bayesian information criterion (26) was used to assess model “goodness of fit” when comparisons between models was necessary. Five percent significance levels and 95% confidence intervals were adopted throughout. Information on confounders and outcomes was complete, so no imputation was needed. All analyses were carried out with STATA 14.1 software (27).

In sensitivity analyses, dose-related risks of the outcomes were estimated for each chronic inflammatory disease, and mixed-effect models were used to account for variation of risk at the general practice level. Kaplan-Meier estimates of outcomes were also calculated for patients with prescribed nonoral glucocorticoids at baseline.

The protocol for the study was approved by the Independent Scientific Advisory Committee for Medicines and Healthcare products Regulatory Agency database research, reference 16_146.

## Results

### Patient characteristics

From 389 general practices, 70,638 eligible glucocorticoid users with one of the six chronic inflammatory diseases were identified. At study entry, the mean age was 59.0 (SD, 17.1) years, and 65.6% were women. Ninety percent of the patients were white (Table 1). During follow-up, prednisolone was the most commonly prescribed drug (98.0% of patients).

**Table 1.** Patient Baseline Characteristics

Characteristics	Oral GC Users (N = 70,638)	Oral GC Nonusers (N = 41,166)
Sociodemographic information		
Age, mean (SD), y	59 (17.1)	52 (18.0)
Men, n (%)	24,476 (34.7)	15,149 (36.8)
Ethnicity, n (%)^*a*^		
White	63,406 (89.8)	34,331 (83.4)
Black	598 (0.9)	598 (1.5)
Asian	1618 (2.3)	1546 (3.8)
Index of multiple deprivation, n (%)		
First (least deprived)	12,543 (17.8)	7221 (17.5)
Fifth (most deprived)	11,378 (16.1)	7282 (17.7)
Body mass index, mean (SD), kg/m^2^	27.07 (5.6)	26.42 (5.8)
Chronic inflammatory disease, n (%)^*b*^		
Inflammatory bowel disease	16,598 (23.5)	15,709 (38.2)
Polymyalgia rheumatica and/or giant cell arteritis	35,007 (49.6)	5007 (12.2)
Rheumatoid arthritis	19,995 (28.3)	15,674 (38.1)
Systemic lupus erythematosus	2748 (3.9)	2678 (6.5)
Vasculitis	4367 (6.2)	3785 (9.2)
Biomarkers, median (IQR)		
C-reactive protein, mg/L	6 (12)	5 (8.3)
Erythrocyte sedimentation rate, mm/h	17 (24)	13 (21)
Comorbidities, n (%)		
Asthma	18,217 (25.8)	5836 (14.2)
Cancer	14,412 (20.4)	5526 (13.4)
Diabetes mellitus	4152 (5.9)	2637 (6.4)
Heart failure	11,489 (16.3)	3334 (8.1)
Chronic kidney disease stage 3 to 4	2211 (3.1)	564 (1.4)
Nonoral prescribed GC in the last y, n (%)		
Inhaled	8248 (11.7)	2120 (5.2)
Intramuscular	422 (0.6)	263 (0.6)
Nasal	4491 (6.4)	2040 (5.0)
Topical	1604 (2.3)	830 (2.0)
Rectal	3099 (4.4)	1848 (4.5)
Prescribed daily oral PED at follow-up start, mean (SD), mg	17.6 (13.8)	NA

Abbreviations: GC; glucocorticoid; IQR, interquartile range; NA, not applicable; PED, prednisolone-equivalent dose.

^a^ Other ethnicity (0.6%) and missing (6.3%).

^b^ Diagnosed before or after the start of follow-up. Patients could have more than one disease.

Polymyalgia rheumatica and/or giant cell arteritis was the most common underlying inflammatory diseases (49.6%), followed by rheumatoid arthritis (28.3%). At baseline, 25.8% of patients had been diagnosed with asthma and 20.4% had cancer. Inhaled and nasal glucocorticoids had been prescribed in the year prior to entry for 16.7% and 6.4% of the patients, respectively. The overall cumulative oral glucocorticoid dose received by patients during follow-up was 5701 mg (1531 mg per year). The highest overall cumulative doses were received by patients with systemic lupus erythematosus (10,049 mg) and vasculitis (9923 mg) (Table 2). During follow-up, adrenocorticotropic hormone and cortisol measurements were recorded for fewer than 1% of patients.

**Table 2.** Timing of Diagnosis of Underlying Chronic Inflammatory Diseases and Description of Oral Glucocorticoid Exposure During the Study Period

Chronic Inflammatory Disease	Diagnosed at Baseline	Diagnosed During Follow-Up	Overall Cumulative Exposure (mg)	Past-y Cumulative Exposure (mg)
IBD	7912 (50.9)	7627 (49.1)	4001 (52.8)	503 (8.2)
PMR-GCA	7374 (22.8)	24,991 (77.2)	6049 (33.8)	910 (6.7)
Rheumatoid arthritis	7838 (46.1)	9120 (53.8)	6315 (65.9)	934 (9.5)
SLE	1210 (54.7)	1001 (45.3)	6834 (199.8)	982 (27.6)
Vasculitis	959 (26.9)	2606 (73.1)	6329 (155.5)	966 (23.9)
All diseases	25,293 (35.8)	45,345 (64.2)	5701 (27.2)	831 (4.5)

Timing of diagnosis data are shown as n (%); cumulative exposure data are shown as mean (SD) PED.

Abbreviations: IBD, inflammatory bowel disease; PED, prednisolone-equivalent dose; PMR-GCA, polymyalgia rheumatica and/or giant cell arteritis; SLE, systemic lupus erythematosus.

### Adrenal insufficiency

There were 183 patients (0.3%) with adrenal insufficiency diagnosed over 450,816 person-years of follow-up. Seventy-one percent of diagnoses were recorded during hospital admissions and 4.9% in the mortality registry (Table 3). The median follow-up time per patient was 5.5 years (range, 1.0 month to 19.3 years), and the overall incidence rate was 0.41 (95% CI: 0.35 to 0.47) per 1000 person-years (Table 4) (15). The crude incidence rate increased with higher daily dose (from 0.30 to 0.86 per 1000 person-years for periods of nonuse and for periods with ≥7.5 mg/d, respectively) and cumulative dose (from 1.91 to 30.48 for <960 and ≥3055 mg/y, respectively, in the past year). Kaplan-Meier risk estimates at 5 years were 0.9% (95% CI: 0.6% to 1.4%) for <960 mg and 17.0% (95% CI: 11.7% to 24.4%) for ≥3055 mg in the past year (15).

**Table 3.** Information on Hospitalization, Endocrinologist Care, and Data Source of Diagnosis for Patients With Adrenal Dysfunction

	Adrenal Insufficiency (N = 183)	Cushing Syndrome (N = 248)	Total^*a*^
Source of diagnosis recording^*a*^			
Primary care	54 (29.5)	199 (80.2)	NA
Hospital admission	130 (71.0)	50 (20.2)	NA
Mortality registry	9 (4.9)	0 (0)	NA
Hospitalized on date of diagnosis or within previous 30 d	150 (82.0)	67 (27.0)	216 (50.6)
Endocrinologist care on the date of diagnosis or within previous 4 mo	6 (3.2)	2 (0.8)	8 (1.9)
Hospitalized in previous 30 d or endocrinologist care in previous 4 mo	152 (83.0)	68 (27.4)	219 (51.3)

Data are presented as n (%).

Abbreviations: NA, not applicable.

^a^ Diagnoses may be recorded in more than one data source.

**Table 4.** Observation Time, Overall Incidence Rates, and Time-Variant Oral Glucocorticoid Prednisolone-Equivalent Dose–Related Incidence Rates of Outcomes

	Adrenal Insufficiency	Cushing Syndrome	Mortality
Total person-y of follow-up	450,816	449,936	451,146
Total incident cases, n (%)	183 (0.3)	248 (0.4)	22, 317 (31.6)
Time at risk per subject, median (IQR), y	5.53 (7.06)	5.52 (7.07)	5.54 (7.07)
Incidence rates per 1000 person-y (95% CI)			
Overall	0.41 (0.35–0.47)	0.55 (0.49–0.62)	49.47 (48.82–50.12)
Daily oral dose			
Nonuse period	0.30 (0.25–0.37)	0.28 (0.23–0.35)	47.27 (46.55–48.01)
>0 to 4.9 mg	0.60 (0.39–0.94)	0.33 (0.18–0.60)	36.09 (34.10–-38.20)
5.0–7.4 mg	0.58 (0.35–0.99)	0.63 (0.38–1.04)	61.57 (58.50–64.79)
≥7.5 mg	0.86 (0.65–1.15)	2.37 (1.99–2.83)	66.38 (64.23–68.61)
Overall cumulative dose			
>0 to 959.9 mg	0.08 (0.05–0.16)	0.21 (0.14–0.31)	30.43 (29.46–31.43)
960–3054.9 mg	0.21 (0.14–0.30)	0.64 (0.52–0.80)	42.61 (41.48–43.76)
≥3055 mg	0.71 (0.61–0.84)	0.69 (0.58–0.81)	64.68 (63.59–65.78)
Cumulative dose in past y			
>0 to 959.9 mg	1.91 (1.37–2.66)	1.86 (1.33–2.60)	272.45 (265.00–280.11)
960–3054.9 mg	9.50 (7.88–11.47)	11.77 (9.94–13.93)	743.19 (727.56–759.17)
≥3055 mg	30.48 (22.27–41.71)	60.90 (48.78–76.03)	1,817.94 (1744.80–1894.15)

Past y means 1 y before the end of follow-up. Total time at risk was calculated as the time between the start of follow-up (when all the eligibility criteria were met) and the end of follow-up (first occurrence of the outcome analyzed, de-registration from practice, or last date of data collection in the practice, whichever happened first).

Abbreviation: IQR, interquartile range.

The risk of adrenal insufficiency increased in periods with higher levels of daily and cumulative glucocorticoid exposure (Table 5). The respective increases in adjusted hazard ratios (HRs) were 1.07 (95% CI: 1.04 to 1.09), 1.09 (95% CI: 1.08 to 1.10), and 2.25 (95% CI: 2.15 to 2.35) for every increase of 5 mg of daily dose, 1000 mg of overall cumulative dose, and 1000 mg of cumulative dose in the past year.

**Table 5.** Time-Variant Prescribed Prednisolone-Equivalent Dose of Oral Glucocorticoids and the Risks of Adrenal Insufficiency, Cushing Syndrome, and Death

	Hazard Ratios With 95% CI		
	Adrenal Insufficiency^*a*^	Cushing Syndrome^*a*^	Mortality^*b*^
Current dose per 5 mg/d	1.07 (1.04–1.09)	1.09 (1.08–1.11)	1.06 (1.05–1.06)
Current dose category (ref: nonuse period)			
>0 to 4.9 mg	2.10 (1.29–3.40)	1.20 (0.64–2.25)	0.63 (0.59–0.67)
5.0–7.4 mg	1.94 (1.11–3.41)	2.07 (1.20–3.57)	1.03 (0.98–1.09)
≥7.5mg	2.95 (2.07–4.21)	6.64 (5.03–8.78)	1.20 (1.16–1.25)
Overall cumulative dose (per 1000 mg)	1.09 (1.08–1.10)	1.10 (1.08–1.11)	1.03 (1.03–1.04)
Overall cumulative dose category (ref: >0 to 959.9 mg)			
960–3054.9 mg	2.75 (1.32–5.74)	4.24 (2.68–6.69)	1.19 (1.14–1.24)
≥3055 mg	14.16 (7.25–27.64)	11.00 (6.95–17.43)	1.64 (1.57–1.71)
Cumulative dose for the past y (per 1000 mg)	2.25 (2.15–2.35)	2.31 (2.23–2.40)	2.05 (2.04–2.06)
Cumulative dose category for the past y (ref: >0 to 959.9 mg)			
960–3054.9 mg	4.98 (3.40–7.30)	6.83 (4.67–9.99)	2.65 (2.56–2.75)
≥3055 mg	15.38 (9.72–24.35)	37.03 (24.58–55.78)	6.66 (6.34–7.00)

All estimates were obtained among glucocorticoid users during the study period.

^a^ Estimates adjusted for inhaled, intra-articular, intramuscular, nasogastric, topical, and rectal use of glucocorticoids.

^b^ Estimates additionally adjusted for asthma, cancer, heart failure, chronic kidney disease, and inflammatory diseases (time-variant).

### Cushing syndrome

There were 248 patients (0.4%) with Cushing syndrome during 449,936 person-years of follow-up. Eighty percent of diagnoses were recorded in primary care and 20.2% during hospital admissions. The median follow-up time was 5.5 years (range, 1 month to 19.3 years), and the overall incidence rate of Cushing syndrome was 0.55 per 1000 person-years (95% CI: 0.49 to 0.62). The crude incidence rate increased with higher daily dose (from 0.28 to 2.37 per 1000 person-years for nonuse and ≥7.5 mg, respectively) and cumulative dose (from 1.86 to 60.90 per 1000 person-years for <960 and ≥3055 mg in the past year, respectively). Kaplan-Meier risk estimates at 5 years were 1.1% (95% CI: 0.7% to 1.6%) for <960 mg and 47.1% (95% CI: 38.6% to 56.5%) for ≥3055 mg in the past year (15).

The risk of Cushing syndrome increased in periods with higher levels of daily and cumulative exposure. The increases in adjusted HRs were 1.09 (95% CI: 1.08 to 1.11), 1.10 (95% CI: 1.08 to 1.11), and 2.31 (95% CI: 2.23 to 2.40) for every increase of 5 mg per day, 1000 mg of the overall cumulative dose over the study period, and 1000 mg of past year cumulative dose, respectively.

### Mortality

#### Patients with prescribed oral glucocorticoids

A total of 22,317 patients (31.6%) with prescribed oral glucocorticoids during the study period died over 451,146 person-years of follow-up. The median follow-up period was 5.5 years (range, 1 month to 19.3 years). Overall, the majority of patients died of cardiovascular diseases (32.4%), followed by cancer (21.0%) and infection (13.3%) (Table 6). Similar patterns were seen for all underlying chronic inflammatory diseases.

**Table 6.** Recorded Causes of Death in Patients With and Without Prescribed Oral Glucocorticoids

Recorded Cause of Death	Patients Prescribed Oral Glucocorticoids	Patients Without Prescribed Oral Glucocorticoids
Primary cause in all patients	N = 22,317	N = 7544
Cardiovascular disease	7235 (32.4)	2437 (32.3)
Cancer	4691 (21.0)	1457 (19.3)
Infection	2958 (13.3)	945 (12.1)
Renal disease	354 (1.6)	117 (1.6)
Respiratory disease	456 (2.0)	149 (2.0)
Other	6623 (29.8)	2469 (32.7)

Data are presented as n (%) unless otherwise noted.

Among patients with prescribed oral glucocorticoids, mortality rates were similar in patients with and without adrenal dysfunction (Table 7). However, patients with adrenal dysfunction had higher mortality rates than patients who did not receive oral glucocorticoids during follow-up (incidence rate ratio = 2.09; 95% CI: 1.64 to 2.63 and incidence rate ratio = 2.24; 95% CI: 1.84 to 2.69 for adrenal insufficiency and Cushing syndrome, respectively). The mortality rate increased with higher daily dose (from 36.09 to 66.38 per 1000 person-years for periods of >0 to 4.9 mg and ≥7.5 mg, respectively) and cumulative dose (from 272.5 to 1818.0 per 1000 person-years for <960 and ≥3055 mg in the past year, respectively). The increases in adjusted HR for every increase of 5 mg per day, 1000 mg of overall cumulative dose, and 1000 mg of past year cumulative dose were 1.06 (95% CI: 1.05 to 1.06), 1.03 (95% CI: 1.03 to 1.04), and 2.05 (95% CI: 2.04 to 2.06), respectively.

**Table 7.** Observation Time, Incidence Rates, and Incidence Rate Ratios of Death During the Study Period

Population Subgroup	Number of Deaths	Person-y of Follow-Up	IRR Compared With Patients Prescribed Oral Glucocorticoids	IRR Compared With Patients Not Prescribed Oral Glucocorticoids
All patients with prescribed oral GCs (N = 70,638)	22,317	451,146	NA	2.05 (1.99–2.10)
Patients with prescribed oral GCs not presenting with adrenal adverse events (N = 70,211)^*a*^	22,132	447,656	NA	2.06 (2.00–2.11)
Patients with oral GC–induced Cushing syndrome (N = 248)	112	2072	1.09 (0.90–1.32)	2.24 (1.84–2.69)
Patients with oral GC–induced adrenal insufficiency (N = 183)	74	1464	1.02 (0.80–1.28)	2.09 (1.64–2.63)
Patients not prescribed oral GCs (N = 41,166)	7544	312,049	—	—

Values in parentheses in the last two columns indicate 95% CIs.

Abbreviations: GC, glucocorticoid; IRR, incidence rate ratio; NA, not applicable.

^a^ Excluding patients who had adrenal insufficiency (n = 183) or Cushing syndrome (n = 248) during follow-up; four patients had both diagnoses recorded.

A total of 74 patients (40.4%) died among patients with oral glucocorticoid-induced adrenal insufficiency, contributing 1464 person-years of follow-up. Fifty percent of these deaths occurred within 11 months after diagnosis (15). Only 13 (15.6%) had their cause of death recorded as adrenal insufficiency. Infection was recorded as the primary or auxiliary cause of death in 44.6% of patients (15). Among patients with oral glucocorticoid-induced Cushing syndrome who contributed 2072 person-years of follow-up, 112 (45.2%) died. Infection was recorded as the primary or auxiliary cause of death in 5.4% of these patients. Fifty percent of these deaths occurred within 36 months after diagnosis.

#### Patients without prescribed oral glucocorticoids

During 312,049 person-years of follow-up, a total of 7544 patients (18.3%) died. The majority died of cardiovascular diseases (32.3%), followed by cancer (19.3%) and infection (12.1%).

### Sensitivity analyses

**Figure 3.**
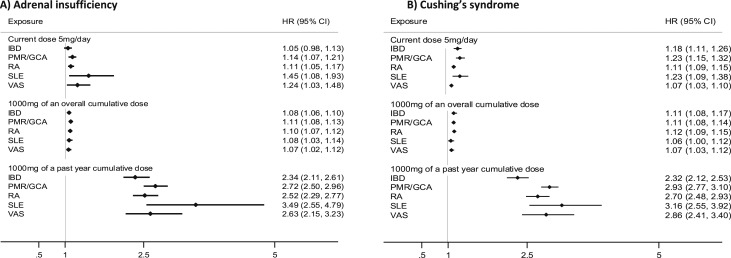
Time-variant cumulative and current dose-related oral glucocorticoid hazard ratios with 95% CIs for adrenal dysfunction by type of chronic inflammatory disease. (A) Adrenal insufficiency. (B) Cushing syndrome. Estimates are adjusted for prescribed inhaled, intra-articular, intramuscular, nasogastric, topical, and rectal glucocorticoids. HR, hazard ratio; IBD, inflammatory bowel disease; PMR/GCA, polymyalgia rheumatica and/or giant cell arteritis; RA, rheumatoid arthritis; SLE, systemic lupus erythematosus; VAS, vasculitis.

**Figure 4.**
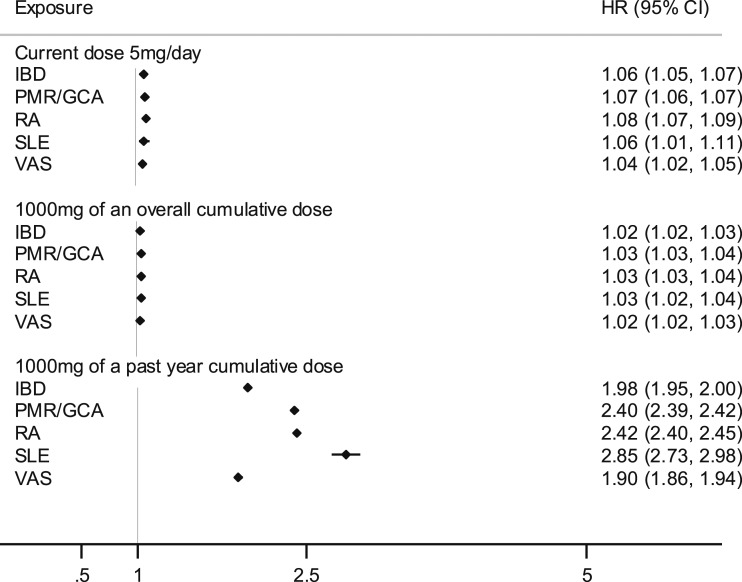
Time-variant cumulative and current dose-related oral glucocorticoid hazard ratios for death by type of chronic inflammatory disease. Estimates are adjusted for prescribed inhaled, intra-articular, intramuscular, nasogastric, topical, and rectal glucocorticoids; baseline asthma; cancer; heart failure; chronic kidney disease; and inflammatory diseases (time-variant). All estimates were obtained among glucocorticoid users during the study period. HR, hazard ratio; IBD, inflammatory bowel disease; PMR/GCA, polymyalgia rheumatica and/or giant cell arteritis; RA, rheumatoid arthritis; SLE, systemic lupus erythematosus; VAS, vasculitis.

Variations in dose-related risks of the outcomes were examined according to the patient’s underlying chronic inflammatory disease (Figs. 3 and 4). The estimates of risk for all diseases were highest for cumulative dose exposure in the last year and for current daily dose. The estimates of death were highest for people with systemic lupus erythematosus. Estimates from mixed and fixed-effect models were similar except for the higher estimates of Cushing syndrome for the cumulative dose in the last year (adjusted HR = 2.53; 95% CI: 2.38 to 2.67) (15).

## Discussion

In this large population-based, retrospective cohort study of 70,638 adults with chronic inflammatory diseases typically treated with oral glucocorticoids, we found a moderate incidence of but strong glucocorticoid dose-response for adrenal insufficiency, Cushing syndrome, and mortality. Specifically, for every increase in daily dose of 5 mg, the risks increased by 7% for adrenal insufficiency, 9% for Cushing syndrome, and 6% for mortality. Likewise, a twofold increase or more in risk of all outcomes was found for every increase in past year cumulative dose of 1000 mg. The patterns of risk were similar for the six diseases investigated, with higher point risk estimates of adrenal adverse events observed in people with systemic lupus erythematosus. The risk of death was twice as high in patients with glucocorticoid-induced adrenal dysfunction as in those not prescribed glucocorticoids during the study period and in glucocorticoid users compared with nonusers during the study period.

Previous studies have reported that glucocorticoid use is associated with higher incidence of adrenal insufficiency (1, 8, 10) and Cushing syndrome symptoms (11) in adults. However, to our knowledge, no study has investigated the relationships between oral glucocorticoid dose and these clinically relevant outcomes.

According to findings from a meta-analysis including 3753 patients from 74 studies, 48.7% of patients with a history of oral glucocorticoid use developed adrenal insufficiency (1). More recent studies conducted in secondary and tertiary care also have reported higher prevalence estimates of adrenal insufficiency in patients who are receiving glucocorticoid therapy (8, 28). However, the incidence rates of adrenal insufficiency and Cushing syndrome in our cohort were extremely low, 0.41 and 0.55 per 1000 person-years, respectively. This most likely reflects a lack of awareness of adrenal insufficiency in primary care and failure to code patients taking glucocorticoids as having secondary Cushing syndrome. Other potential reasons for this disparity could be differences in case ascertainment and in the type and severity of underlying diseases considered, resulting in lower glucocorticoid exposure. Our study reflected routine clinical practice, included patients with six common chronic inflammatory diseases, and used diagnosis data recorded by physicians in primary care and during hospital admissions. In contrast, the published meta-analysis used clinical data from patients with allergic diseases, cancer, and organ transplantation; defined adrenal insufficiency according to serum cortisol levels; and had a mean daily prednisolone-equivalent dose at the start of follow-up of 30 to 90 mg (compared with 17 mg in our cohort).

To minimize misclassification of non‒glucocorticoid-induced events in our cohort, we used a highly stringent definition of glucocorticoid-induced adrenal dysfunction through implementation of an algorithm based on diagnoses recorded in primary and hospital care and the mortality registry and prescribed medications. Episodes of adrenal insufficiency diagnosed and treated in accident and emergency admissions but not requiring hospitalization are therefore likely to have been excluded from our study if general practitioners did not code the information in patient records. Furthermore, 71% of diagnoses were recorded during hospital admissions or at death, and 83% of all cases of adrenal insufficiency occurred within 1 month of hospitalization or shortly after a consultation with an endocrinologist; in addition, of 1343 patients receiving hydrocortisone during follow-up, only 77 had a diagnosis of oral glucocorticoid-induced adrenal insufficiency recorded. This suggests that underdiagnosis and underrecording of adrenal dysfunction is common in clinical practice. Indeed, in our study, fewer than 1% of the 70,638 patients receiving oral glucocorticoids had adrenocorticotropic hormone and cortisol testing results recorded in primary care. In addition, among patients who stopped taking glucocorticoids before the end of follow-up and did not restart the treatment (81% of patients), the incidence of mortality following cessation of oral glucocorticoid therapy markedly increased during the first 2 months after cessation and then rapidly decreased over time after the first 3 months of interruption (15). This is consistent with the existence of episodes of undiagnosed adrenal crisis in clinical practice leading to avoidable deaths.

Adrenal crisis remains a life-threatening complication of glucocorticoid deficiency. Even in educated patients with a diagnosis of primary and secondary adrenal insufficiency, the incidence ranged between five and 10 crises per 100 person-years, with a 6% crisis-associated mortality rate (29, 30). Our data highlight the critical need for increased patient and health practitioner awareness of the real risk of adrenal crisis in this situation—its clinical features, prevention, and treatment. It is now timely to create and implement clear diagnosis and management pathways to improve clinical monitoring of patients treated with long-term glucocorticoid therapy.

The incidence of death found among glucocorticoid users in our cohort is similar to the 23.4% estimate reported in another study of patients with rheumatoid arthritis treated with oral glucocorticoids that used a similar methodology (12), but dose-response estimates were lower. However, it is likely that some deaths caused by adrenal insufficiency (*e.g.,* among patients with episodes of acute illness such as infections) were missed and were not reported as related to adrenal insufficiency. This is also supported by the observed increased mortality rate following treatment interruption that rapidly decreased 3 months after cessation.

The study has other limitations. First, although prescribed medications are automatically recorded in the CPRD, minimizing recording errors, the end date of the prescriptions was not available in the data. This date was therefore estimated from information recorded on the product prescribed and the directions given to the patient on how to take the drug; if missing, it was imputed to estimate the period covered by the prescription. Second, our data did not include prescriptions issued by specialists in hospitals. We may have therefore underestimated the dose taken by patients during the period around diagnosis or at times of disease flare. Third, we used oral glucocorticoid prescription as a proxy for the actual intake of the drugs. However, prescriptions reflect the actual dose taken only if adherence is optimal. Fourth, although we implemented strict algorithms to identify glucocorticoid-induced adrenal dysfunction, misclassification of diagnosis cannot be completely ruled out.

Nonetheless, our study has important strengths. First, we used linked electronic health records that spanned primary and hospital care and were linked to the mortality registry in England. Second, the sample size was large and representative, allowing us to estimate risks more precisely and improve generalizability, respectively. Third, oral glucocorticoid dose was standardized (*i.e.,* converted to prednisolone-equivalent dose), minimizing risk of bias due to within- and between-patient variations in prescribed glucocorticoids. Fourth, prescribed medications are automatically recorded in the CPRD, minimizing recording errors. Fifth, to minimize length and time-dependent bias, patient exposure status was taken into account during analyses. Sixth, to minimize bias due to confounding and overadjustment, we systematically selected confounding variables and used statistical tools (directed acyclic graphs) to address the problem. Seventh, in addition to estimating glucocorticoid dose‒related risks of death among patients treated with oral glucocorticoids during the study period, mortality was also compared between oral glucocorticoid users and nonusers among patients diagnosed with the same chronic inflammatory diseases.

In conclusion, in this study we quantified glucocorticoid dose‒dependent risks of adrenal insufficiency, Cushing syndrome, and death not previously reported. Increased rates were still observed in patients taking the equivalent of 5 mg of prednisolone per day. The observed low incidence of reported adrenal dysfunction in patients taking oral glucocorticoids and the increase in mortality following treatment interruption suggest lack of awareness and need for both design and implementation of clear diagnosis and management pathways and for patient and health practitioner education to prevent avoidable deaths.

## Abbreviations:

CPRD: Clinical Practice Research Datalink
HR: hazard ratio
NIHR: National Institute for Health Research
